# Acute Sleep-Wake Cycle Shift Results in Community Alteration of Human Gut Microbiome

**DOI:** 10.1128/mSphere.00914-19

**Published:** 2020-02-12

**Authors:** Zhi Liu, Zhi-Yuan Wei, Junyu Chen, Kun Chen, Xuhua Mao, Qisha Liu, Yu Sun, Zixiao Zhang, Yue Zhang, Zhou Dan, Junming Tang, Lianhong Qin, Jian-Huan Chen, Xingyin Liu

**Affiliations:** a State Key Laboratory of Reproductive Medicine, Nanjing Medical University, Nanjing, China; b Department of Pathogen-Microbiology Division, Key Laboratory of Pathogen of Jiangsu Province, Nanjing Medical University, Nanjing, China; c Key Laboratory of Human Functional Genomics of Jiangsu Province, Nanjing Medical University, Nanjing, China; d Key Laboratory of Holistic Integrative Enterology, Second Affiliated Hospital of Nanjing Medical University, Nanjing, China; e Department of Clinical Laboratory, Affiliated Yixing People’s Hospital, Jiangsu University, Wuxi, China; f Laboratory of Genomic and Precision Medicine, Department of Public Health and Preventive Medicine, Wuxi School of Medicine, Jiangnan University, Wuxi, China; University of Michigan—Ann Arbor

**Keywords:** circadian rhythm, gut microbiome, human disease, microbial interaction, sleep-wake cycle shift

## Abstract

Disturbances of sleep and the underlying circadian rhythm are related to many human diseases, such as obesity, diabetes, cardiovascular disorders, and cognitive impairments. Dysbiosis of the gut microbiome has also been reported to be associated with the pathologies of these diseases. Therefore, we proposed that disturbed sleep may regulate gut microbiota homeostasis. In this study, we mimicked the sleep-wake cycle shift, one typical type of circadian rhythm disturbances in young people, in recruited subjects. We used 16S rRNA gene amplicon sequencing to define microbial taxa from their fecal samples. Although the relative abundances of the microbes were not significantly altered, the functional-profile analysis of gut microbiota revealed functions enriched during the sleep-wake cycle shift. In addition, the microbial networks were quite distinct among baseline, shift, and recovery stages. These results suggest that an acute sleep-wake cycle shift may exert a limited influence on the gut microbiome, mainly including the functional profiles of the microbes and the microbial relationships within the microbial community.

**IMPORTANCE** Circadian rhythm misalignment due to social jet lag, shift work, early morning starts, and delayed bedtimes is becoming common in our modern society. Disturbances of sleep and the underlying circadian rhythms are related to multiple human diseases, such as obesity, diabetes, cardiovascular disorders, and cognitive impairments. Given the crucial role of microbiota in the same pathologies as are caused by sleep disturbance, how the gut microbiota is affected by sleep is of increasing interest. The results of this study indicate that the acute circadian rhythm disturbance caused by sleep-wake shifts affect the human gut microbiota, especially the functional profiles of gut microbes and interactions among them. Further experiments with a longer-time-scale intervention and larger sample size are needed to assess the effects of chronic circadian rhythm disruption on the gut microbiome and to guide possible microbial therapies for clinical intervention in the related diseases.

## Abstract (precis)

Circadian rhythm misalignment due to social jet lag, shift work, early morning starts, and delayed bedtimes is becoming common in our modern society. Disturbances of sleep and the underlying circadian rhythms are related to multiple human diseases, such as obesity, diabetes, cardiovascular disorders, and cognitive impairments. Given the crucial role of microbiota in the same pathologies as are caused by sleep disturbance, how the gut microbiota is affected by sleep is of increasing interest. The results of this study indicate that the acute circadian rhythm disturbance caused by sleep-wake shifts affect the human gut microbiota, especially the functional profiles of gut microbes and interactions among them. Further experiments with a longer-time-scale intervention and larger sample size are needed to assess the effects of chronic circadian rhythm disruption on the gut microbiome and to guide possible microbial therapies for clinical intervention in the related diseases.

## INTRODUCTION

Circadian rhythm is a defining feature of mammalian metabolism that synchronizes metabolic processes to day-night light cycles (1). This rhythm aligns biological functions with regular and predictable environmental patterns to optimize function and health. Circadian rhythm misalignment due to chronic sleep loss, social jet lag, shift work, early morning starts, and delayed bedtimes is becoming common in our modern society (2). Chronic sleep deficiency is linked to a multitude of health conditions; for example, shift workers who change their sleep-wake cycle frequently are more likely to gain weight and suffer from hypertension and diabetes (3, 4) and even cancers (5, 6). Disturbances of both sleep and the underlying circadian rhythm also resulted in many neurological and psychiatric diseases, such as Alzheimer disease (AD) (7). Even an acute period of sleep-wake cycle disturbance can cause changes in glucose metabolism, hormone production, and weight gain (3, 4). Cedernaes et al. reported that acute sleep loss can result in tissue-specific alterations in the genome-wide DNA methylation state and metabolic fuel utilization in humans (8). A recent study has revealed that acute sleep loss may have detrimental effects on brain health even in younger individuals by increasing tau protein, the AD-associated plasma biomarker (9).

Dysbiosis of the gut microbiome is known to be associated with diabetes, metabolic disorders, and fat storage (10, 11), as well as impaired cognitive performance through the gut-brain axis (12–14). Given the crucial role of microbiota in the same pathologies caused by sleep disturbance, how the gut microbiota is affected by sleep is of increasing interest. Two studies focusing on the effect of sleep loss on gut microbiota were conducted. In one study involving nine male volunteers, two nights of partial sleep deprivation of 4 h (02:45 to 07:00) significantly decreased insulin sensitivity compared to the insulin sensitivity after normal sleep (22:30 to 07:00) during baseline. The abundances of a few gut microbiota species were changed during the short-term sleep loss (15). However, a longer-term study on 11 participants, which included two episodes of five nights of sleep restriction (04:00 to 08:00), separated by five nights of 12 h of sleep (22:00 to 10:00), with a final 12-h sleep recovery for two nights, did not show changes in the fecal microbial composition at baseline, during sleep restriction, or after sleep recovery (2). These studies set a uniform bedtime and wakeup time artificially for all of the subjects, which ignored the personal idiosyncracies of sleep patterns, i.e., each of the subjects might have a unique accustomed sleeping time and sleep duration. Thus, the observed outcome could have resulted not only from the sleep loss but also a possible sleep-wake cycle shift.

In this study, we aimed at exploring the effect of a sleep-wake cycle shift, which is common in students and shift workers like nurses and flight attendants, on the human gut microbiome. We recruited 22 volunteers aged 20 to 35 years. The subjects were required to keep their regular routines for at least 7 days before the experiment and then to postpone their regular sleeping time for 2 to 4 h and wake up naturally the next morning to mimic the sleep-wake cycle shift. The fecal samples were collected at three time points: 1 day before the experiment (T1), the morning after the night of the sleep time shift (T2), and 2 days after they returned to their regular sleep time (T3). 16S rRNA gene amplicon sequencing was applied to these samples to characterize the microbiota alterations from the acute circadian rhythm shifts. A taxon-by-taxon analysis indicated no statistically significant alteration of gut microbiota among the three time points. However, the functional-profile analysis of gut microbiota revealed functions enriched at T2. In addition, network analysis revealed that the communities within the microbiota were significantly changed. Together, these results show that acute sleep-wake cycle shift is unlikely to induce large-scale shifts in the composition of the microbiome; however, the microbiota functional profile and community relationships are disturbed over the time scales studied.

## RESULTS

### The overall change of gut microbiota during acute circadian rhythm shifts.

**FIG 1:**
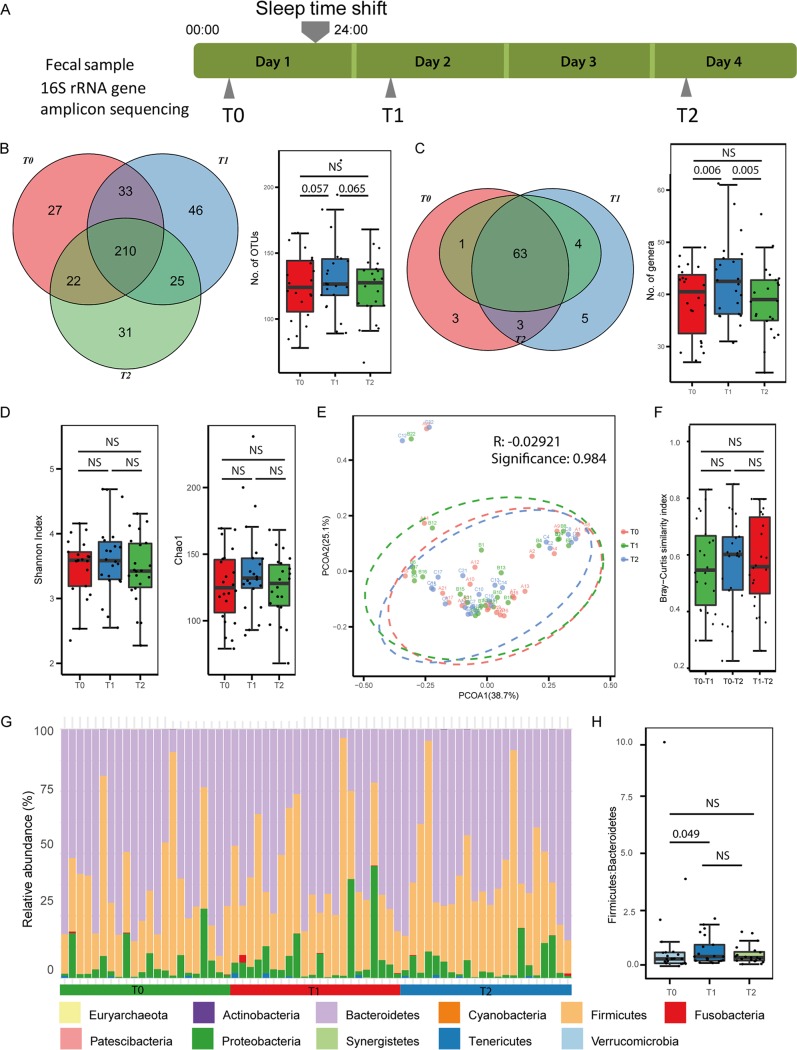
Overview of the study and the gut microbiome. (A) Summary of the experimental design. (B) Venn diagram (left) and box plot (right) of the numbers of OTUs identified at baseline (T0), after the sleep-wake cycle shift (T1), and at recovery (T2). Dots in box plot show values in each individual. (C) Venn diagram (left) and box plot (right) of the numbers of genera identified at T0, T1, and T2. A significantly increased number of genera is observed after the sleep-wake cycle shift. Dots in box plot show values in each individual. (D) Alpha diversity values of the gut microbiomes at T0, T1, and T2. (E) Beta diversity values of the gut microbiomes at T0, T1, and T2. PCOA, principal coordinate axis. (F) Distribution of Bray-Curtis similarity index values between T0 and T1, T0 and T2, and T1 and T2. Dots in box plot show values in each individual. (G) Relative abundances of phyla in samples. (H) *Firmicutes/Bacteroidetes* ratios of gut microbiomes at T0, T1, and T2. NS, not significant.

Fecal samples were collected by the 22 study subjects 1 day before the experiment, to determine the baseline microbiome (baseline, T0), on the morning after the sleep-wake cycle shift (shift, T1), and 2 days after the subjects returned to their regular routine (recovery, T2) (Fig. 1A). The samples were sequenced by 16S rRNA gene amplicon sequencing, and 1,671 microbiota operational taxonomic units (OTUs) were identified by using Qiime2 (16). By comparing the OTUs that were detected repeatedly (detected in more than 2 samples) in the three time points, we observed only a marginally significant difference in the numbers of OTUs between baseline and shift (Mann-Whitney U test, *P *= 0.057) and between shift and recovery (Mann-Whitney U test, *P *= 0.065) (Fig. 1B). The OTUs were then collapsed to the genus level, and a significantly higher number of detectable genera (with at least 10 reads in no fewer than 3 samples at each time point) was observed at shift compared with the numbers at both baseline (Mann-Whitney U test, *P *= 0.006) and recovery (Mann-Whitney U test, *P *= 0.005). The number of genera at the recovery stage had decreased and was comparable to the baseline level (Fig. 1C). This result indicated a tendency for the number of genera to increase after the sleep time shift; however, this increase was quickly reversed after participants returned to their normal routines (recovery stage) (Fig. 1C). The alpha diversity was not significantly different among the three groups (Fig. 1D). Principal coordinate analysis based on the weighted UniFrac index exhibited no significant difference in bacterial composition among groups (Fig. 1E). Additionally, we calculated the Bray-Curtis similarity values between T0 and T1, T0 and T2, and T1 and T2 in each individual, and no significant differences were observed (Fig. 1F). The phyla *Bacteroidetes*, *Firmicutes*, and *Proteobacteria* dominated across the three time points (Fig. 1G). Given that changes in the ratios of the phyla *Firmicutes* and *Bacteroidetes* (F/B) have been associated with obesity (17) and type 2 diabetes (11), we compared the F/B ratios among any two time points. An increase in the F/B ratio at T1 (after sleep-wake time shift) compared with that at T0 (baseline) was observed (paired, one-sided Mann-Whitney U test, *P* = 0.049) (Fig. 1H).

### Abundances and alteration in function of gut microbes during acute circadian rhythm shifts.

**FIG 2:**
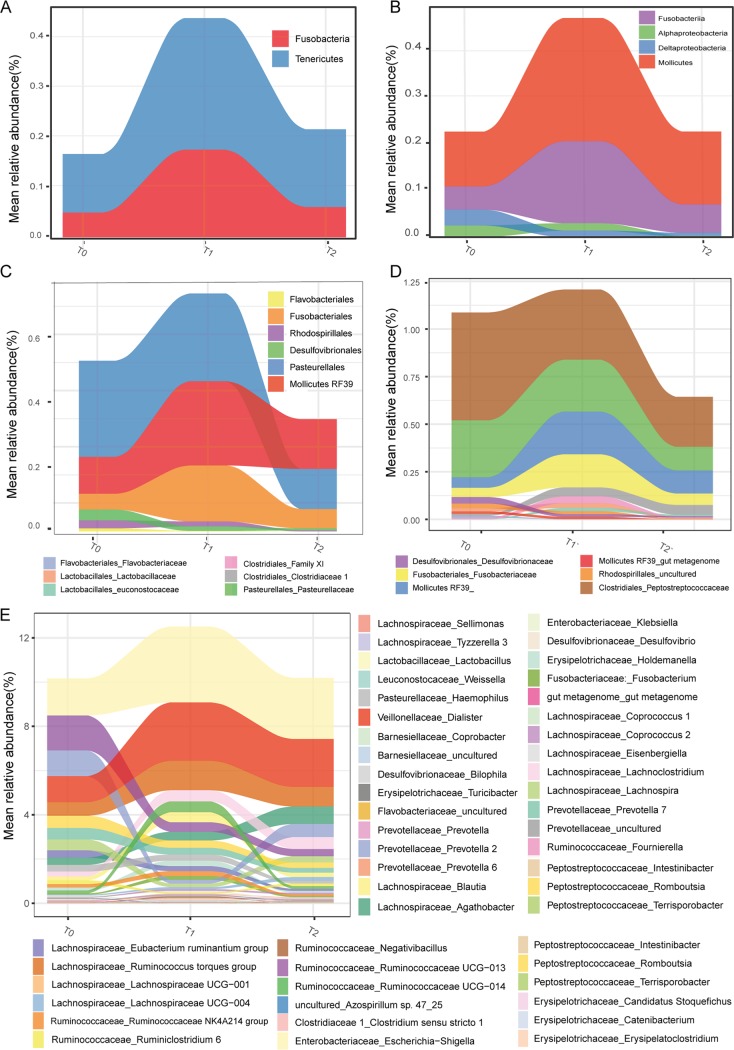
Alteration in abundances of microbes at different phylogenetic levels. The changes of microbial relative abundances from T0 to T1 and then to T2, presented at phylum (A), class (B), order (C), family (D), and genus (E) level.

There were no significant changes in taxa observed by statistical test among the baseline, shift, and recovery samples in our data set, which is in accordance with the results from a previous study of sleep restriction in rats and humans (2). Thus, we focused on the microbes whose average relative abundance changed more than 2-fold between any two of the time points to characterize the tendency of alteration, instead of identifying statistically differential abundance. Distinct patterns in changes of abundance were observed (Fig. 2). The phyla *Fusobacteria* and *Tenericutes* and classes *Fusobacteriia* and *Mollicutes* were increased after sleep shift and decreased to be comparable to the baseline level at recovery (Fig. 2A and B), representing the taxa that are resilient in response to acute sleep-wake cycle shift. The order *Pasteurellales* and family *Clostridiales_Peptostreptococcacea* gradually decreased from baseline to recovery, indicating a long-term impact of acute sleep-wake shift on them (Fig. 2C and D).

**FIG 3:**
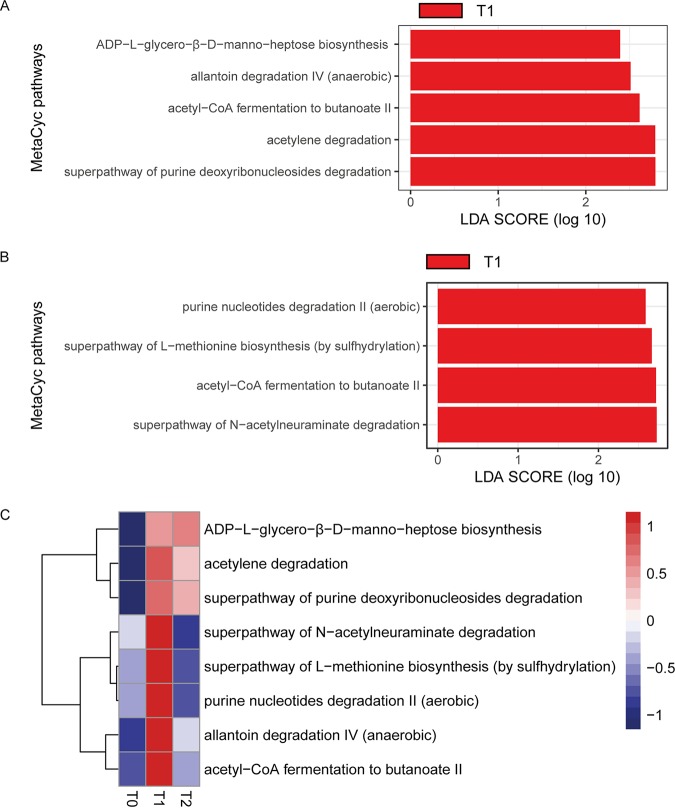
Alteration of predicted microbial functions between different time points. (A) Differential MetaCyc pathways between T0 and T1 identified by LEfse. LDA, linear discriminant analysis. (B) Differential MetaCyc pathways between T1 and T2 identified by LEfse. (C) Heatmap of the mean abundances of all the differential pathways across three time points.

Next, to explore the alteration in function of the fecal microbiota at different time points, PICRUSt2 (phylogenetic investigation of communities by reconstruction of unobserved states) was used to infer the abundances of the MetaCyc pathways from the 16S rRNA gene amplicon sequencing. There was no alteration in pathways between time points T0 and T2. However, in the comparison between T0 and T1, five pathways were significantly enriched at T1, including ADP-l-glycero-β-d-manno-heptose biosynthesis, allantoin degradation IV (anaerobic), acetyl-coenzyme A (CoA) fermentation to butanoate II, acetylene degradation, and the superpathway for purine deoxyribonucleoside degradation (Fig. 3A). Four pathways were decreased at T2 compared with their abundances at T1, i.e., purine nucleotide degradation II (aerobic), the superpathway for l-methionine biosynthesis (by sulfhydrylation), acetyl-CoA fermentation to butanoate II, and the superpathway for *N*-acetylneuraminate degradation (Fig. 3B). Collectively, purine metabolism was increased during acute circadian rhythm shifts. Deregulated purine metabolism has been reported in multiple diseases, including gout, colitis, autism, and Alzheimer disease (AD) (18–21). The pathway for acetyl-CoA fermentation to butanoate II was significantly increased at T1 and significantly decreased at the recovery stage. Butanoate metabolism is related to short-chain fatty acid (SCFA) metabolism, which has been reported to be involved in energy metabolism (22), inflammation (23), and psychological functioning (24) of the host. Additionally, we also observed that most of the pathways that were increased at T1 had a tendency to be decreased at T2, though not statistically significantly (Fig. 3C).

### Alteration of microbiome interaction underlying acute circadian rhythm shifts.

**FIG 4:**
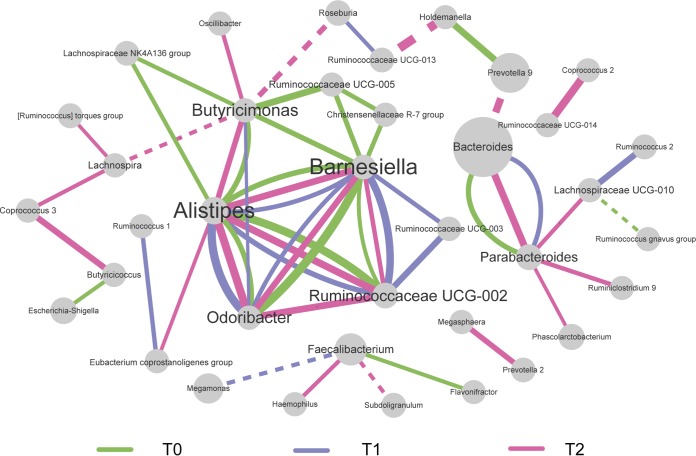
Microbial interactions over time. Cooccurrence network of microbes at genus level. The threshold of SparCC correlations was *r* ≥ 0.6. Green, purple, and pink lines represent the connections of two genera at baseline (T0), after the sleep-wake cycle shift (T1), and at recovery (T2), respectively. Dotted and solid lines indicate negative correlation and positive correlation, respectively. The thickness of the line is proportional to the correlation value. The sizes of the nodes are proportional to their relative abundances.

Inspired by the idea that a slight change in abundance can introduce microbiome structural alteration (25), analysis of the cooccurrence network of genera at the three time points was conducted. The correlations with an *r* of ≥0.6 are shown in Fig. 4. The cooccurrences among *Alistipes*, *Barnesiella*, and *Odoribacter* were preserved at baseline (T0), shift (T1), and recovery (T2), and the interaction between *Bacteroides* and *Parabacteriodes* remained intact during the acute circadian rhythm shifts. Notably, the cooccurrences between *Butyricimonas* and other genera were mainly observed at T0 and T2 but not at T1. Members of *Butyricimonas* are butyric acid-producing bacteria. Butyric acid is an important energy source for intestinal epithelial cells and plays a role in the inflammatory process and maintenance of colonic homeostasis (26). A previous study in mice has implicated circadian rhythm disturbance as affecting the intestinal microbiota, which may have implications for inflammatory diseases (27). However, the taxa that cooccurred with *Butyricimonas* were different at T0 and T2, suggesting the existence of alterations in microbiome organization even after the return to the regular routine.

**FIG 5:**
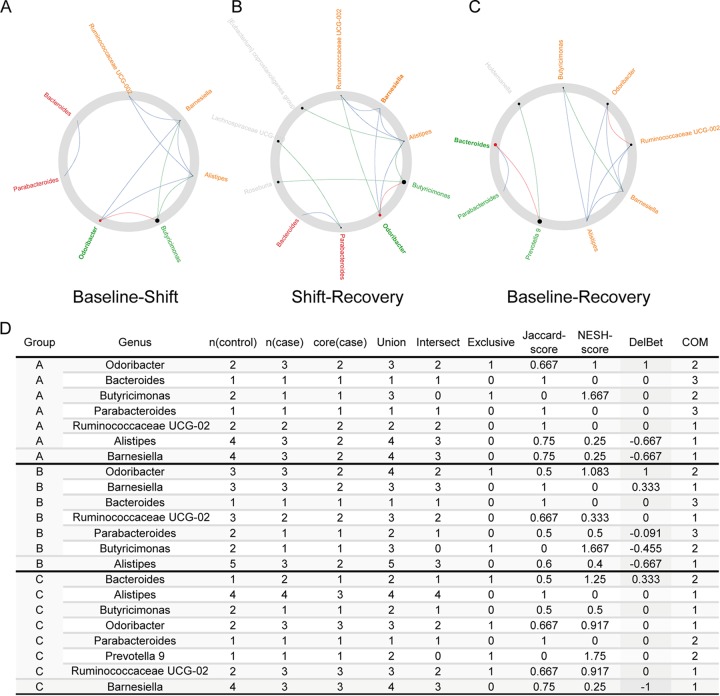
The network drivers between each two time points. (A to C) The network drivers that drive network organization changes from baseline to shift (A), from shift to recovery (B), and from baseline to recovery (C). Red nodes represent the drivers identified by the NetShift method. The sizes of nodes are proportional to the NESH scores. (D) Summary of the node properties calculated by NetShift.

Next, the NetShift method (28) was used to analyze the driver genera that hold key significance in microbial interactions. The genus *Odoribacter* was predicted as a driver of the community change from T0 to T1 (Fig. 5A and D). *Odoribacter* is a producer of short-chain fatty acids like acetate, butyrate, and propionate (29), and studies have reported that increased *Odoribacter* may partly contribute to decreased inflammation (30) and may be associated with colon cancer (31). In addition, *Odoribacter* was also predicted to drive the community change from T1 to T2 (Fig. 5B and D), suggesting that the acute circadian rhythm shifts may introduce transient alteration in the gut microbiome interaction network through *Odoribacter*. *Bacteroides* is predicted as a driver to promote community change between T0 and T2 (Fig. 5C and D). *Bacteroides* is a well-known genus that is implicated in metabolic diseases, including obesity and diabetes (32, 33). For example, the depletion of species from the *Bacteroides* genus in obese individuals was proposed to be related to the higher concentrations of aromatic amino acids and branched-chain amino acids in circulation, which are known risk factors for type 2 diabetes and cardiovascular disease (34). This result suggested that the acute sleep-wake cycle shift may exert an effect on the interactions between gut microbes even after the return to the normal routine.

## DISCUSSION

Circadian rhythm misalignment due to chronic sleep loss, social jet lag, shift work, early morning starts, and delayed bedtimes is becoming common in our modern society. Disturbances of sleep and the underlying circadian rhythms are related to many human diseases, such as obesity, diabetes, cardiovascular disorders, cognitive impairments, and cancers. A prospective cohort study that followed 143,410 nurses for 22 to 24 years indicated that rotating night shift work was associated with a higher risk of type 2 diabetes, with a hazard ratio of 1.31 (95% confidence interval, 1.19 to 1.44) (35). Flight attendants are another typical population suffering from frequent circadian rhythm disruption. Studies on flight attendants reveal a higher risk of breast cancer than in the general population (5, 6). Though many studies attribute this to the higher exposure to cosmic radiation, circadian rhythm disruption is proposed as an important risk factor (5, 6). The development of breast cancer has been linked to disruption of circadian rhythms, which is usually caused by shift work, short sleep duration, and exposure to light at night (36, 37). The underlying mechanism of breast cancer is reported to be related to hormonal alteration, which could also result from circadian rhythm disturbance (38). Additionally, circadian rhythm disruption was reported to lead to significant changes in the immune system and biological processes associated with breast cancer risk (39). A meta-analysis showed a significant association between breast cancer risk in women and circadian rhythm-disrupting exposures when aggregating all studies, regardless of the source of circadian rhythm disruption (odds ratio, 1.21; 95% confidence interval, 1.08 to 1.21) (37). Furthermore, significantly increased tumor growth was observed in an animal study of mice that simulated chronic-jet-lag-caused circadian rhythm disruption (40).

Previous studies (2, 15) have shown only slight or even no alteration of gut microbial abundance and diversity during sleep intervention. This might be due to short times of intervention in circadian rhythm and small sample sizes. In this study, we included a relatively larger sample size and focused on the sleep-wake shift by mimicking the natural situation where people change the sleep-wake cycle but not necessarily the sleep duration. Consistent with the previous studies, we only observed slight changes in microbial abundance and diversity. However, the functional profile prediction analysis of gut microbiota revealed several functions enriched at T2; for example, the purine metabolism pathways, the deregulation of which has been reported in multiple diseases, including colitis (19), autism spectrum disorder (20), and AD (21). A newly published study has reported the effect of acute sleep loss on the increase of an AD-risk biomarker (9). Our results suggest possible new ways in which sleep disturbance may be involved in neurodevelopmental disorders. In addition, acetyl-CoA fermentation to the butanoate II pathway, which is related to SCFA metabolism, is also enriched at T2. An abundance of evidence has shown that SCFAs influence multiple biological processes, including energy metabolism (22), psychological functioning (24), immune and inflammatory responses (23), and others. A newly published study reported the role of SCFAs in modulating recovery after experimental stroke via immunological mechanisms (41).

Furthermore, considering that the human microbiome is a complex bacterial community where specific interactions between individual microbes play important roles in their functionality and the maintenance of eubiosis (42–44), we explored the network changes that occurred during the sleep intervention. Distinct network structures were observed among the time points of baseline (T0), intervention (T1), and recovery (T3) (Fig. 4), and the driver genus that drove the network structure alteration from baseline to intervention and from intervention to recovery was the same, i.e., *Odoribacter* (Fig. 5A and B). In addition, although the diversity and abundances of microbes in the recovery stage (T2) were similar to those in the baseline (T0), *Bacteroides* drove the network structure changes between them (Fig. 5C). These results indicated that the acute circadian rhythm disturbance caused by sleep-wake shifts affected the human gut microbiota mainly though microbial functionality and the interactions of gut microbiota.

However, it remains an open question whether the gut microbiome will recover and how long it will take, and thus, experiments with a longer-time-scale intervention and larger sample size are needed to assess the effects of chronic circadian rhythm disruption on gut microbial abundances, as well as network organization.

## MATERIALS AND METHODS

### Ethics statement.

This study was approved by the Ethics Committee of Yixing People’s Hospital of Jiangsu University (ethics no. 2016005). All participants were provided a written informed consent upon enrollment. Once the consent forms were signed, we screened them for eligibility criteria and sent questionnaires and sample collection kits to participants.

### Study subject recruitment.

Twenty-two healthy adults aged from 22 to 35 years were recruited from Nanjing Medical University and Yixing People’s Hospital (Table S1). All subjects had received no antibiotics, probiotics, prebiotics, or antifungal medications for 3 months prior to fecal sample collection, and none of them were on anti-inflammatory or antioxidant drugs. The participants were asked to maintain their regular lifestyle throughout the study, including the timing and type of meals, except for the sleeping time as we required.

10.1128/mSphere.00914-19.1 TABLE S1 Information about subjects. Download Table S1, XLSX file, 0.01 MB. Copyright © 2020 Liu et al. 2020 Liu et al. This content is distributed under the terms of the Creative Commons Attribution 4.0 International license.

### Fecal sample collection and DNA extraction.

Fecal samples of each subject were obtained at home as directed in the instructions provided and frozen immediately until delivered to the laboratory. Fecal samples were delivered together with dry ice and were stored at –80°C at the laboratory until extraction.

About 100 mg of sample was weighed in the laboratory from each fecal sample and used to extract total-genome DNA, following the protocol of the DNA extraction kit (product number DP328; Tiangen Company, Beijing, China). The concentration and purity of the extracted bacterial DNA were detected using a Qubit 2.0 fluorometer (Thermo Scientific, USA). DNA quality and quantity were determined by agarose gel electrophoresis.

### 16S rRNA gene amplicon sequencing and analysis.

The genomic DNA in fecal samples was extracted using the DNA extraction kit (product number DP328; Tiangen Biotech Co., Ltd., Beijing, China). The DNA concentration was measured using a Qubit 2.0 fluorometer (Thermo Fisher Scientific, USA). The 16S rRNA gene amplification procedure was divided into two PCR steps. In the first PCR, the hypervariable V4 region of the 16S rRNA gene was amplified using the conserved primers 515F (5′-GTGCCAGCMGCCGCGGTAA-3′) and 806R (5′-GGACTACHVGGGTWTCTAAT-3′). Then, amplification was performed in 96-well microtiter plates. Reactions were run with the following program: 2 min of denaturation at 94°C, followed by 30 cycles of 20 s at 94°C (denaturing), 30 s at 56°C (annealing), and 40 s at 68°C (elongation), with a final extension at 68°C for 5 min. The PCR products were quantified using the Quant-iT PicoGreen quantification system (Life Technologies), and samples with a concentration above 6 ng/μl were diluted to ∼3 to 6 ng/μl prior to further analysis. In the second PCR step, sequencing primers and adaptors were added to the amplicon products. The PCR was run according to the cycling program described above except with a reduced cycle number of 15. The amplification products were purified with Agencourt AMPure XP beads (Beckman Coulter Genomics, MA, USA) according to the manufacturer’s instructions. Equimolar amounts of the amplification products were pooled in a single tube. The pooled DNA samples were concentrated using the DNA Clean & Concentrator-5 kit (ZymoResearch, Irvine, CA, USA) according to the manufacturer’s instructions. The concentration of the pooled libraries was determined using the Quant-iT high-sensitivity DNA assay kit (Life Technologies) following the specifications of the manufacturer. Amplicon sequencing was performed on the Illumina HiSeq system (BGI, China). Automated cluster generation and 2 × 250-bp paired-end sequencing with dual-index reads were performed. The sequencing output was generated as demultiplexed fastq-files for downstream analysis.

Bioinformatics analysis of 16S rRNA gene amplicons was performed by using Qiime2 (version 2018.6.0) (16). Briefly, fastq reads were processed by using the dada2 program, and the dada2 denoise-paired command was used to delete the low-quality ones. Dada2 generates unique features that could be compared between different studies. The taxonomy of these features was assigned via the Greengenes reference database (version 13-2) classifier with 99% similarity. Determinations of alpha and beta diversities were also conducted in Qiime2.

### Function prediction of gut microbiome.

The functional capacity of the gut microbial community was predicted using PICRUSt2. The OTU table generated from Qiime2 was rarefied according to the lowest sample sequence (10,214) and then supplied to PICRUSt2. Predicted functional genes were categorized into MetaCyc pathways (45) and compared across each of the two time points using LEfse. Statistical differences in MetaCyc frequencies were determined with the default parameters (linear discriminant analysis [LDA] score of >2).

### Network analysis.

The SparCC algorithm (46) was used to estimate the correlations from the compositional network. The network was demonstrated by cytoscape with edges connecting nodes (bacterial taxa) with a correlation coefficient of over 0.6 or less than −0.6. NetShift (28) analyses were conducted in the web server using the networks generated by SparCC.

### Data availability.

All raw 16S rRNA gene sequencing data have been deposited in the NCBI Sequence Read Archive (SRA) under accession number SRP227414.
